# Impact of Oral Supplementation of Different Levels of Tamoxifen on Productive and Reproductive Efficiencies and Carcass Traits of Avian48 and Arbor Acres Broilers

**DOI:** 10.3390/ani10081367

**Published:** 2020-08-06

**Authors:** Mona E.M. Younis, Asmaa Aboelnour, Ayman A. Swelum, Hanan A. Ghoneim, Mohamed E. Abd El-Hack, Asmaa F. Khafaga, Muath Q. Al-Ghadi, Ahmed R. Al-Himadi, Bader O. Almutairi, Aiman A. Ammari, Mahmoud M. Abo Ghanima

**Affiliations:** 1 Department of Animal Husbandry and Animal Wealth Development, Faculty of Veterinary Medicine, Damanhour University, Damanhour 22511, Egypt; 2 Department of Histology and cytology Faculty of Veterinary Medicine, Damanhour University, Damanhour 22511, Egypt; 3 Department of Animal Production, College of Food and Agriculture Sciences, King Saud University, P.O. Box 2460, Riyadh 11451, Saudi Arabia; 4 Department of Theriogenology, Faculty of Veterinary Medicine, Zagazig University, Zagazig 44511, Egypt; 5 Department of Physiology, Faculty of Veterinary Medicine, Damanhour University, Damanhour 22511, Egypt; 6 Poultry Department, Faculty of Agriculture, Zagazig University, Zagazig 44511, Egypt; 7 Department of Pathology, Faculty of Veterinary Medicine, Alexandria University, Edfina 22758, Egypt; 8 Department of Zoology, College of Science, King Saud University, Riyadh 11451, Saudi Arabia; 9 Department of Veterinary Medicine, College of Agriculture and Veterinary Medicine, Thamar University, Dhamar 87246, Yemen

**Keywords:** Tamoxifen, broiler breeds, performance, carcass, oestrogen, gonads

## Abstract

**Simple Summary:**

The effect of oral supplementation of Tamoxifen on productive and reproductive efficiencies and carcass characteristics of two broiler breeds was investigated in the current study. Tamoxifen supplementation can improve growth performance and carcass efficiency of broilers without changing the sex hormonal profile, however, treatment of broiler chicken with Tamoxifen in different doses caused a gradual decrease in follicle production rate and eventually led to an increase of the atretic follicles in different stages of atresia. Further research is required to estimate the best concentration required for each breed.

**Abstract:**

This research was aimed at estimating the effect of oral supplementation of Tamoxifen on productive efficiency, carcass characteristics, hormonal profile and gonadal structure of two broiler breeds. One hundred and eighty chicks of each breed of Avian48 and Arbor Acres were divided into three groups: control group; TAM10 group, supplied with 10 mg Tamoxifen/kg of body weight at 3, 5, 7 and 9 days of life; and TAM20 group, supplied at the same intervals with 20 mg Tamoxifen/kg of body weight. Both levels of Tamoxifen improved productive performance at early ages, but Arbor Acres produced better results with TAM20 levels than TAM10, while Avian48 breeds reacted adversely. On the contrary, Tamoxifen supplementation significantly decreased feed intake and feed conversion (after the first two weeks of life) compared to control with a higher level of decrease reported for TAM20 treatments than TAM10 and for Arbor Acres compared to Avian48 breed. Carcass traits were not affected significantly with Tamoxifen supplementation compared to control although Arbor Acres responded better to TAM20 and Avian48 for TAM10. With regard to the effect of Tamoxifen (TAM) on sex hormones, it could be concluded that TAM10 treatments showed a stimulating effect on the level of such hormones as compared with the TAM20 group with the most favourable results being clearly detectable in 42-day-old birds although both concentrations of Tamoxifen did not differ significantly from control. However, treatment of broiler chickens with Tamoxifen in different doses caused a gradual decrease in follicle production rate and eventually led to an increase of the atretic follicles in different stages of atresia. Finally, we can conclude that Tamoxifen supplementation can improve performance and carcass efficiency of broilers without changing the hormonal profile, however much research is required to estimate the best concentration required for each breed.

## 1. Introduction

Oestrogen plays an important role in bird sex differentiation, which is essential for the growth of ovaries and the regulation of the proliferation of the left gonadal cortex [1]. In addition, 17b-Hydroxysteroid dehydrogenase and aromatase enzymes that are responsible for converting androgens to estradiol-17b are detectable only in the gonads of female embryos [2]. Oestrogens cause their effects in target cells through oestrogen receptors (ERs) located not only in the ovary and oviduct but also in different tissues including the pituitary gland and hypothalamus [3]. Many studies have been applied to induce sex reversal from female to male by suppression of oestrogen secretion to obtain the benefits of unisex males. Tamoxifen (TAM), which is one of the aromatase inhibitors, induces a wide range of activity in different species [4]. TAM has been suggested not to be a pure oestrogenic antagonist, but it has both antiestrogenic and oestrogenic effects, depending on the dose given [5]. Low doses of TAM advanced ovarian and oviductal development, increased oestrogen and androgen in plasma, and induced early egg laying in hens [6,7]. In the contrast, the administration of high doses of TAM caused blockage of oestrogen receptors leading to a progressive decrease in the laying of eggs before their complete cessation [8]. TAM‘s impact at different ages has been studied, either by ova inoculation to estimate its influence on sex determination as a modulator for oestrogen receptors [9,10,11,12] or hatch day [9] or even older ages to assess its impact on hormonal profile and reproductive system [6,13,14]. Additionally, the various administration techniques of Tamoxifen were studied: in-ovo inoculation [9], per os in gelatine capsules [15] and intra muscular injection as well as the use of different preparation doses, e.g., 0.5; 1.0; 5.0; 10.0 or even 25 mg/kg body weight. However, the effect of Tamoxifen supplementation on performance of different broiler breeds has not been studied.

Therefore, the present study was conducted to investigate the effect of early oral Tamoxifen administration at different doses on growth performance, carcass characteristics, hormonal profile and histological structure of sex organs of two different broiler breeds up to 42 days of age.

## 2. Materials and Methods

### 2.1. Birds and Experimental Design

The present study was conducted at a research center for poultry production, the Faculty of Veterinary Medicine of the Damanhour University. The experimental protocol regarding the care and handling of animals was approved by the Native Experimental Animal Care Committee and approved by the Ethics of the Institutional Committee of Animal Husbandry and Animal Wealth Development Department, Faculty of Veterinary Medicine, Damanhour University, Damanhour, Egypt (DMU/VetMed-2019-/0145). On the same day, one-hundred-and-eighty-day-old chicks from each of the Avian48 and Arbor Acres breeds were sourced from local country hatcheries. Chicks from each breed were wing banded at first day of age; then divided into three groups of 60 chicks; chicks of each group were subdivided into 5 replicates (10 chicks/replicate). The groups were 1. Control group, Tamoxifen; 2. TAM10 group in which chicks obtained TAM on the third, fifth, seventh and ninth day of age by oral administration at level of 10 mg per kg of body weight; and 3. TAM20 group in which chicks got 20 mg TAM per kg of body weight at the same time intervals. TAM (Amriya for Pharmaceutical Industries, Alexandria, Egypt) was used as TAM citrate. Chicks were brooded under gas brooders supplying 33 °C at the first week and reduced by 3 °C per week to 24 °C. For the first week of life, illumination was supplied for 24 h then the lighting time was shortened to 18 h a day. Birds were fed a starter ration of 23% crude protein (CP) during the first three weeks of age then provided with a grower ration of 21% CP until the end of experiment at 42 days; ration and water were offered ad libitum during the experimental period. The experimental diets were formulated according to National Research Council (NRC) [16] recommendations. The ingredient content and composition of the diets is shown in Table 1.

### 2.2. Productive Efficiency

The measured productive performance traits including weekly body weight (g), weekly weight gain, weekly feed consumption (g/bird/week) and weekly feed conversion ratio.

### 2.3. Reproductive Efficiency

Reproductive efficiency evaluation included measuring of serum concentrations of sex hormones and histological evaluations of the gonads. Blood samples were collected twice at 35 and 42 days from the wing vein of the same birds. Three birds per replicate were used for blood collection, serum was separated for 15 min by centrifugation at 3000 rpm, and preserved in a deep freezer at −20 °C until the time of analysis. The concentrations of serum testosterone and oestrogen were determined by enzyme-linked immunosorbent assay (ELISA) using a commercial ELISA kit (Wuhan Fine Biotech Co., Ltd., China) according to the manufacturer’s instructions.

Left and right gonads were taken from three males and three females of each treatment following the macroscopic examination. Gonads were sliced into smaller pieces, fixed in 4% paraformaldehyde in 0.1M phosphate buffer saline (PBS) (PH 7.4) overnight at −40 °C. After fixation, tissues were processed for histological examination starting with dehydration in ascending grades of ethyl alcohols, cleared with xylene and embedding with melted paraffin wax. Later, 6-µm-thick sections were dewaxed and stained with hematoxylin and eosin stain. Images were captured using a Nikon DM ×1200 digital camera (Tokyo, Japan) at a magnification of ×20 and ×400, and saved in JPEG format.

### 2.4. Carcass Traits

Carcass traits assessed at 42 days of age after three birds per replicate were selected randomly and deprived of food for 12 h with persistence on water. The selected birds were weighed before slaughtering then reweighed again after evisceration to calculate dressing percentage. Abdominal fat with fat around the gizzard; internal organs (intestine, liver, gizzard and heart), comb and wattle were weighed to the nearest gram using a sensitive scale (0.0000) and calculated relative to their live weight. The carcasses were divided and the thigh, shoulder and breast weight with bone was weighed and calculated relative to carcass weight.

### 2.5. Statistical Analysis

Data were analyzed using a two-way analysis of variance by SAS [17], Proc GLM (*p* < 0.05), significant differences between means were determined by Duncan multiple range test [18].

## 3. Results

### 3.1. Productive Efficiency

**Figure 1:**
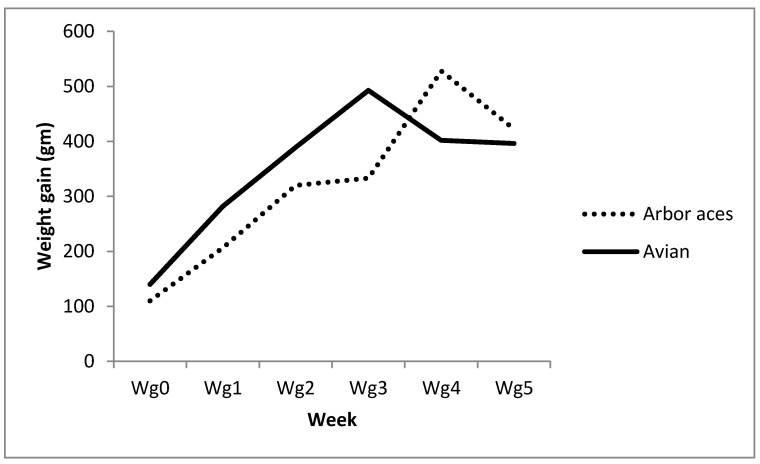
Weekly weight gain of Avian48 and Arbor Acres breeds.

Data in Table 2 show that the Avian48 breed had attained substantially higher body weights over all experimental times than the Arbor Acres’ raises. Moreover, the data reports significantly higher weight gains than Arbor Acres (Table 2) except for the fifth and sixth weeks when the reverse was true. Figure 1 shows that the Avian48 breed had achieved maximum weight gain earlier than the Arbor Acres breed. Oral administration of TAM at various levels increased broilers’ body weight compared to control, but this increase was only significant in the second and third weeks (Table 2). Influence of TAM administration on body weights and weight gains differed according to broiler breed where the Arbor Acres breed responded better to TAM20 than TAM10 and control. However, the Avian48 breed yielded better results with TAM10 than TAM20 and control treatments (Table 2 and Table 3).

With respect to feed intake, the Avian48 breed reported significantly higher feed intake over the entire experiment compared to the Arbor Acres breed (Table 4). TAM administration increased feed intake in the first two weeks of the experiment then it decreased and was significantly lower than control birds until the end, where the lowest total feed intake was reported for TAM20 groups followed by TAM10 and the highest values were for control groups (Table 4).

In general, the feed conversion ratio decreased as birds aged with the lowest values found during the third and fourth week of age (Table 5). With respect to breed impact there was no significant difference between different breeds for total feed conversion ratio. TAM20 induced a higher feed conversion ratio than TAM10 and control groups during the second week of age, however from the third week until the end of the experiment TAM20 contributed to better feed conversion ratios compared to TAM10 and control groups with significant differences reported during the fourth week. With observing the interaction between breed and treatment we found that TAM20 had a better effect on Arbor Acres’ feed conversion than control and TAM10 (especially with increasing age). On the contrary the effect of TAM20 on feed conversion of the Avian48 breed was negative, particularly with increasing age, although total feed conversion ratios were not significantly different between different treatments (Table 5).

### 3.2. Reproductive Efficiency

#### 3.2.1. Serum Sex Hormones Levels

As shown in Table 6, serum testosterone was significantly increased in birds at 42 days of age compared with 35 days of age. Additionally, testosterone concentrations in males were significantly increased compared to females in all groups. All experimental groups also reported significant rises in the level of testosterone at 42 days of age relative to the level at 35 days of age with the highest level in the TAM10 group as compared with the TAM20 group (Table 7). Meanwhile, age, treatment and sex interaction had a significant effect on testosterone hormone with the highest level found in TAM10 males compared to TAM20 males at 42 days of age and the lowest level in female control at 35, 42 days of age (Table 8).

Table 6 shows a significant rise in serum oestrogen concentrations in birds at the age of 42 days, compared to 35 days. In females, the level of oestrogen also increased significantly compared with males of all groups. In addition, females of all groups had a significantly increased oestrogen as compared to males with the highest level found in the TAM10 female group as compared to the TAM20 female group (Table 7). Meanwhile, the interaction between age, sex and treatment had a significant effect on oestrogen hormone with the highest level found in TAM10 females as compared to TAM20 females at 42 days of age and the lowest level present in TAM10, 20 males at 35 days of age and control males at 42 days of age (Table 8).

#### 3.2.2. Gonads Histology

**Figure 2:**
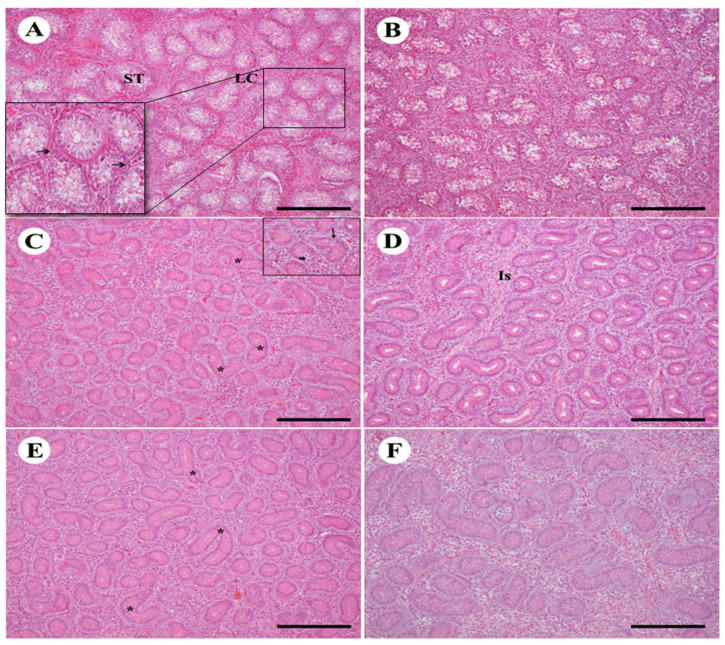
Effect of TAM treatment with different doses on testicular histology of two bird strains. (**A**,**B**): control testis of Avian48 A and Arbor Acer B shows normal structure of seminiferous tubules (ST) and Leydig cells (LC) in between. (**C**,**D**): testes treated with Tam10 showing different shapes of ST deformations in Avian48 C (*), giant cells (small arrow in the inset) and germinal epithelium differentiation (arrow), interstitial tissue Is, in Arbor Acres D. (**E**,**F**): testes treated with Tam20 with more deformities (*) Avian48 **E**, Arbor Acres **F**. H&E stain, scale bar = 100 µm.

Birds treated with TAM had regressive changes in the histology of the testicles. The male bird control testis of both strains showed seminiferous tubules correctly organized with interstitial tissues containing Leydig cells (Figure 2A,B). In the species Arbor Acres, tubules were found more loosely contacted than the Avian48. In the seminiferous tubules, birds treated with low-dose TAM displayed various atrophy characteristics including a major decrease in the diameter of the seminiferous tubule. Testis of Avian48 showed a small rise in the seminiferous tubules with multilayered epithelium and several spermatogenesis stages of in seminiferous tubules started to occur with few Sertoli cells and giant cells (Figure 2C,D). The seminiferous tubules and interstitial tissue were degrading and contained fibrous material. Seminiferous tubules displayed shape deformations: comma and S shape. There was a significant rise in both interstitial tissue and Leydig cells relative to the control group. The same characteristics were demonstrated in the Arbor Acres, however more deformities of the tubules and more fibrous material in the interstitial tissue with decreased germinal epithelium thickness made the lumen widen (Figure 2C,D). High-dose TAM-treated birds showed more deformations in seminiferous tubules of both with decrease in Leydig cells and more fibrous material found in the interstitial tissues that were more obvious in the Arbor Acres. In Arbor Acres, there was also an increase of the thickness of germinal epithelium in seminiferous tubules (Figure 2E,F).

**Figure 3:**
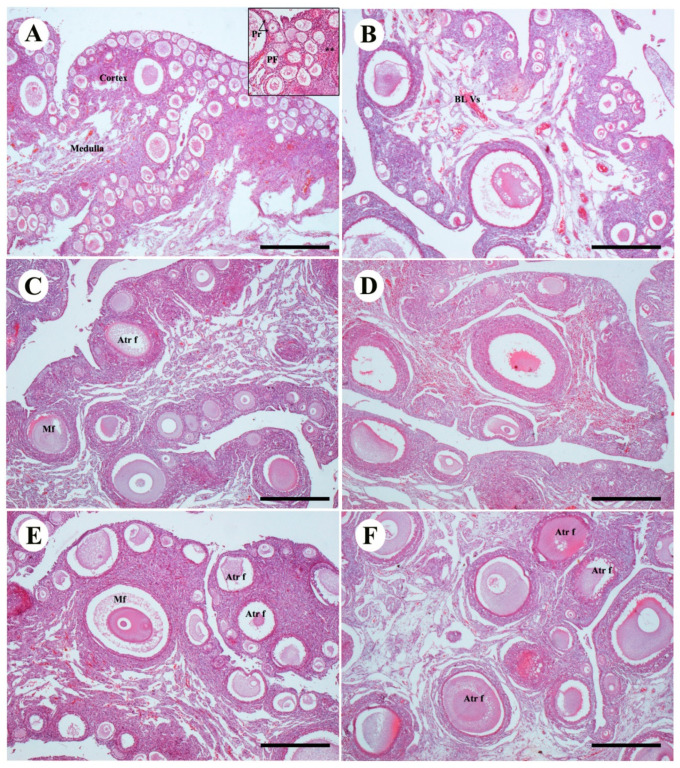
Effect of TAM treatment with different doses on ovarian structures of two bird strains (**A**,**B**): control group of Avian48 A and Arbor Acres B ovaries showing normal follicles of ovarian cortex, primordial follicle pr, primary follicle p and theca cells (astrix). (**C**,**D**): ovaries treated with Tam10 in Avian48 C and Arbor Acres D showing increase in the atretic follicles and decrease in the primary and primordial follicles. (**E**,**F**): ovaries treated with Tam20 of Avian48 E and Arbor Acres F showing different stages of atretic follicles. Atretic follicles Atr f, mature follicles Mf. H&E stain, scale bar 100 µm.

Drug treatment with TAM also had an effect on ovarian structure and histology. Unilateral ovary was present in the control group of females including the formation of follicles. In the ovarian cortex there were immature and mature follicles, composed of oocytes encircled by granulosa cells. Theca cells were also present (Figure 3A,B). In Avian48 the number of mature and immature follicles was greater than in Arbor Acres. The ovarian medulla consisted of medullary vessels and collagen fibers which were more apparent than the cortex vessels. A rise in the developed primary follicles was present in low-dose groups treated with TAM, and few numbers of mature follicles were also demonstrated. Atretic follicles were noticed in Arbor Acres species treated with low-dose TAM (Figure 3C,D). The high-dose TAM treatment exhibited more atresia in the follicles found in both. The number of atretic mature follicles was significantly increased either in early stage of atresia or in the late atretic follicles (Figure 3E,F).

### 3.3. Carcass Traits

The Avian48 breed registered significantly higher carcass weight, dressing percentage, breast percentage and shoulders percentage compared to the Arbor Acres breed (Table 9). Although Arbor Acres’ thigh percentage was higher than the Avian48 breed, Arbor Acres’ thigh percentage was higher than its breast percentage. Oral administration of various levels of TAM did not alter carcass characteristics significantly relative to control treatments although Arbor Acres chickens responded better to TAM20 and the Avian48 breed to TAM10 treatment (Table 9). Arbor Acres chickens had substantially higher percentages of intestine, liver and gizzard (relative to live weight) than the Avian48 breed (Table 10). There were no significant variations between different treatments in the percentages of internal organs.

## 4. Discussion

Tamoxifen exerts mixed antagonistic-agonistic properties, i.e., it shows mammary gland antioestrogenic activity and agonistic effects on the uterus. Nevertheless, it is used to inhibit the adverse effects of oestrogens in endocrine therapy for breast cancer patients [19].

This experiment was designed to determine the effect on performance and carcass traits of different broiler breeds (Avian48 and Arbor Acres) of oral administration treated with different levels of aromatase inhibitor (Tamoxifen) at an early age. The Avian48 breed attained higher body weight, carcass weight, dressing percentage and percentage carcass cuts compared to the Arbor Acres breed except for thigh percentage and giblets weights that were higher in the Arbor Acres breed. Souza et al. [20] found that the breast yield of Ross, Cobb and Hubbard was higher than that of Arbor Acres. In addition, Makram et al. [21] worked on four genetic lines of broiler chicks (Arbor, Avian48, Hubbard and Cobb), recording higher body weight, carcass weight, dressing percentage and percentage edible carcass cuts for the Avian48 breed than the Arbor Acres breed, but thigh percentage and nonedible parts were higher for Arbor Acres than the Avian48 breed.

Oral TAM administration at an early age increased body weight of the two breeds at some weeks but there were no differences at the final weighing. Dewil et al. [22] concluded similar findings that in-ovo inoculation with fadrazole (another azole-type aromatase inhibitor) enhanced body weight at an early age, but this effect faded by the fifth week of age and their results were due to increased levels of GH, T3 and testosterone resulting from aromatase inhibitor injection. In addition, feed intake increased substantially during the first week of the experiment (second week of age) in Tamoxifen treated groups compared to the control administration than control, with TAM20 groups having numerically higher intake than TAM10 groups, however, the TAM20 and TAM10 supplementation subsequently decreased feed intake compared to the control but this occurred more rapidly in TAM20 treatment. As a result, the total feed conversion ratio for chickens under TAM20 treatment was lower than control and even TAM10. Our findings were in contrast to Nahid et al. [12] who concluded that there were no significant differences in bird weight, weight gain, feed consumption and feed-to-gain ratio between treated and control groups, but this disparity could be attributable to drug dosage, injection time, number of chickens injected or strain.

With regard to treatment and breed interaction the Arbor Acres breed responded better to Tamoxifen supplementation particularly TAM20 where it increased final body weight, weight gain and reduced feed intake which significantly improved feed conversion ratio than TAM10 and control treatments. However response of the Avian48 breed was better to TAM10 than TAM20 where early supplementation of Avian48 chickens with Tamoxifen 20 mg/kg of body weight decreased its performance compared to control and TAM10. Further work is needed to explain the impact of Tamoxifen on various breeds of broilers.

Supplementation with Tamoxifen did not significantly affect carcass characteristics compared to control procedures; similar findings were concluded in a previous study by Nahid et al. [12]. While TAM20 increased carcass cuts percentages numerically than TAM10 and control groups in Arbor Acres breed, Avian48 chicken TAM20 given lower carcass characters than TAM10 and control groups. Tamoxifen supplementation did not change the relative percentages of internal organs significantly except for the percentage of the heart, where it was significantly higher than control under TAM10 treatment. Nahid et al. [12] found that Tamoxifen inoculation in-ovo did not significantly affect internal organs as compared to the control.

Oestrogens, secreted from the ovary in the female, or synthesized by testosterone in situ aromatization in the male, exert a negative feedback effect on the secretion of gonadotropins at the level of both brain and adenohypophysis [4]. Feeding of juvenile White Leghorn (WL) female chicks on oestrogenic compounds delayed the onset of egg laying by two to three weeks [23]. On the other hand, antiestrogens can induce gonadotropin secretion and gonadal activity through inhibition of oestrogen binding to its receptors in the brain and pituitary gland [6].

With regard to the effects of both age and sex on testosterone and oestrogen hormones, our results are in agreement with a previous study [22], which reported a significant effect of these two factors on levels of such hormones with significantly higher testosterone levels in males compared to females and vice versa for oestrogen. Age, treatment and sex interaction also had a significant effect on testosterone and oestrogen hormones with the highest levels in 42-day male and female birds, respectively, that received Tamoxifen at a dose of 10 mg/kg of body weight compared to those which received 20 mg/kg of body weight. This result is consistent with previous research [6] which revealed that low doses of TAM improved both oviductal and ovarian development, increased plasma oestrogen and androgen levels with subsequent early egg production in young WL hens. Meanwhile, administration of low doses of TAM to male WL chicks stimulated testicular and comb growth, increased plasma testosterone concentrations with simultaneous precocious semen production and sexual activity. On the other hand, contrary results are produced by high doses of TAM, so it was concluded that TAM has both antiestrogenic and oestrogenic properties in WL male chicks, depending on the dose [5], and is not a pure oestrogen antagonist as suggested previously. Therefore, it can be concluded that TAM’s antiestrogenic effect stimulates gonadotropic activity, but when the dose was high enough, it competes with the elevated oestrogen in circulation, thereby reducing its efficacy in the target tissues [6].

The results showed dose-dependent regressive testicular histological alterations. The results reconfirmed the earlier findings which stated the estradiol had an essential role in spermatogenesis [24]. These observations suggest that reduced oestrogenic action induced by Tamoxifen suppresses spermatogenesis by inhibiting the proliferation and survival of germ cells and by promoting the rate of apoptosis of germ cells. Recent studies on mice treated with Tamoxifen in vivo showed substantial dose-dependent decline in the expression of aromatase enzyme in the testis, which was also associated with sperm decline [25]. In a human study, Robertson et al. [26] indicate that Tamoxifen-mediated decreased aromatase synthesis in the testis may cause decreased synthesis of estradiol endogenously which may in effect be responsible for regressed spermatogenesis. In birds, however, it has been known that Tamoxifen has antagonistic properties—it binds to ER receptors and inhibits the effect of oestrogen in the target tissue [27].

In this study, treatment of broiler chicken with Tamoxifen in different doses caused a gradual decrease in follicle production rate and eventually led to an increase in the atretic follicles at different stages of atresia. This result is consistent with previous findings that have shown that treatment of laying hens with Tamoxifen leads to a reduction in the rate of egg laying and evokes a pause in egg laying [28]. The gradual decrease in the rate of egg laying after Tamoxifen administration is probably associated with the abolition of the central action of oestrogens at the hypothalamo-pituitary axis. It cannot be ruled out that, by blocking the ER receptors in the central nervous system, Tamoxifen prevents the occurrence of the preovulatory surge of LH in response to progesterone [29].

## 5. Conclusions

It could be concluded that Tamoxifen supplementation can improve growth performance and carcass efficiency of broilers without change its sex hormonal profile, however, treatment of broiler chicken with Tamoxifen in different doses caused a gradual decrease in follicle production rate eventually leading to an increase of atretic follicles at different stages of atresia. Further research is required to estimate the best concentration required for each breed.

## Figures and Tables

**Table 1:** Experimental ration composition and ingredients.

Items	Starter Ration	Grower Ration
CP%	22.71	20.46
Energy	3030	3160
Fiber	3.1	2.9
Ca +	0.98	0.82
Phosphorous	0.57	0.50
Methionine	0.65	0.62
Lysine	1.4	1.14
Thyronine	0.95	0.84
Na	0.23	0.20
Chloride	0.23	0.20
NaCl	0.003	0.003

**Table 2:** The effect of breed, Tamoxifen treatments and their interactions on weekly body weight (Means ± SE).

Item		Week0	Week1	Week2	Week3	Week4	Week5	Week6
**Breed Effect**								
Arbor Acres		45.79 ± 0.73 ^a^	153.38 ± 3.84 ^b^	356.8 ± 10.96 ^b^	667.92 ± 17.54 ^b^	1002.8 ± 24.94 ^b^	1531.51 ± 27.96 ^b^	1998.30 ± 35.49 ^b^
Avian48		42.98 ± 0.56 ^b^	182.59 ± 2.95 ^a^	464.33 ± 8.46 ^a^	855.86 ± 13.72 ^a^	1329.33 ± 19.27 ^a^	1728.51 ± 21.61 ^a^	2109.79 ± 26.64 ^a^
**Tamoxifen Effect**								
Control		43.66 ± 0.58	160.46 ± 3.06 ^b^	389.45 ± 8.62 ^b^	741.94 ± 14.2	1126.27 ± 20.13	1632.5 ± 22.57	2075.33 ± 26.79
TAM10		45.75 ± 0.91	171.85 ± 4.79 ^a^	409.3 ± 13.75 ^ab^	761.8 ± 22.00	1189.70 ± 31.12	1647.7 ± 34.9	2074.18 ± 46.22
TAM20		43.75 ± 0.86	171.65 ± 4.51 ^a^	432.93 ± 12.96 ^a^	781.93 ± 20.74	1182.23 ± 29.34	1609.83 ± 32.9	2012.62 ± 39.72
Interaction effect (Breed * Tamoxifen)								
Arbor Acres	Control	44.38 ± 0.83	144.13 ± 4.51 ^b^	331.13 ± 12.55 ^c^	640.50 ± 20.08 ^c^	976.53 ± 29.34 ^c^	1545.47 ± 32.9 ^d^	1985.31 ± 38.46 ^c^
	TAM10	48.00 ± 1.49	156.00 ± 7.82 ^b^	339.6 ± 22.45 ^c^	645.60 ± 35.92 ^bc^	979.20 ± 50.82 ^c^	1498.40 ± 56.99 ^d^	1991.25 ± 76.91 ^bc^
	TAM20	45.00 ± 1.36	160.00 ± 7.14 ^b^	399.67 ± 20.49 ^b^	717.67 ± 32.79 ^b^	1052.67 ± 46.4 ^c^	1550.67 ± 52.02 ^cd^	2018.33 ± 62.8 ^bc^
Avian48	Control	42.94 ± 0.81	176.78 ± 4.12 ^a^	447.78 ± 11.83 ^a^	843.38 ± 20.08 ^a^	1276.00 ± 27.56 ^b^	1719.53 ± 30.91 ^ab^	2165.35 ± 37.31 ^a^
	TAM10	43.50 ± 1.05	187.70 ± 5.53 ^a^	479.00 ± 15.88 ^a^	878.00 ± 25.4 ^a^	1400.20 ± 35.94 ^a^	1797.00 ± 40.30 ^a^	2157.11 ± 51.28 ^ab^
	TAM20	42.50 ± 1.05	183.30 ± 5.53 ^a^	466.20 ± 15.88 ^a^	846.20 ± 25.4 ^a^	1311.80 ± 35.94 ^ab^	1669.00 ± 40.30 ^bc^	2006.90 ± 48.65 ^bc^
*p-value*								
Breed		0.002	<0.000	<0.000	0.003	0.012	0.002	0.004
Tamoxifen		0.562	0.012	<0.000	0.085	0.069	0.856	0.095
Breed × Tamoxifen		0.856	0.003	0.001	0.041	0.020	0.001	0.015

^a,b,c,d^ Mean within the same column with different superscripts are significantly different (*p* < 0.05).

**Table 3:** The effect of breed, Tamoxifen treatments and their interactions on weekly weight gain (Means ± SE).

Items		WG0	WG1	WG2	WG3	WG4	WG5	Total gain
**Breed Effect**								
Arbor Acres		110.16 ± 3.94 ^b^	206.42 ± 9.77 ^b^	319.93 ± 15.36 ^b^	333.04 ± 18.57 ^b^	527.83 ± 17.35 ^a^	422.40 ± 23.54	1952.67 ± 36.07 ^b^
Avian48		139.76 ± 2.9 ^a^	281.73 ± 7.54 ^a^	389.07 ± 11.42 ^a^	492.68 ± 14.35 ^a^	401.60 ± 13.71 ^b^	396.11 ± 19.07	2072.10 ± 26.91 ^a^
**Tamoxifen Effect**								
Control		117.67 ± 3.07 ^b^	233.50 ± 7.69	348.80 ± 11.64	410.38 ± 14.99	495.58 ± 14.39	473.43 ± 19.53 ^a^	2039.25 ± 26.86
TAM10		126.10 ± 4.72 ^ab^	237.45 ± 12.25	352.50 ± 18.56	427.90 ± 23.18	458.00 ± 21.53	421.55 ± 30.42 ^ab^	2029.04 ± 46.97
TAM20		131.10 ± 4.72 ^a^	261.28 ± 11.55	362.20 ± 18.56	400.30 ± 21.85	440.56 ± 20.72	408.78 ± 27.55 ^b^	1968.87 ± 40.37
Interaction Effect (Breed * Tamoxifen)								
Arbor Acres	Control	101.07 ± 4.60 ^d^	196.00 ± 11.19 ^c^	309.38 ± 16.94 ^b^	330.53 ± 21.85 ^b^	566.29 ± 21.01 ^a^	464.93 ± 28.52	1940.94 ± 39.09
	TAM10	108.00 ± 7.70 ^cd^	183.60 ± 20.01 ^c^	306.00 ± 30.30 ^b^	333.60 ± 37.85 ^b^	519.20 ± 35.16 ^a^	424.6 ± 47.72	1943.75 ± 78.17
	TAM20	121.40 ± 7.70 ^bc^	239.67 ± 18.27 ^b^	344.4 ± 30.30 ^ab^	335.00 ± 34.56 ^b^	498.00 ± 32.10 ^ab^	467.67 ± 43.56	1973.33 ± 63.83
Avian48	Control	134.28 ± 4.06 ^ab^	271.00 ± 10.55 ^ab^	388.22 ± 15.97 ^a^	490.24 ± 20.53 ^a^	424.88 ± 19.66 ^bc^	481.94 ± 26.68	2137.56 ± 36.85
	TAM10	144.20 ± 5.45 ^a^	291.30 ± 14.15 ^a^	399.00 ± 21.43 ^a^	522.20 ± 26.77 ^a^	396.80 ± 24.86 ^c^	417.50 ± 37.73	2114.33 ± 52.11
	TAM20	140.80 ± 5.45 ^a^	282.90 ± 14.15 ^a^	380.00 ± 21.43 ^a^	465.60 ± 26.77 ^a^	383.11 ± 26.21 ^c^	349.90 ± 33.74	1964.40 ± 49.44
*p-value*								
Breed		0.001	0.002	0.002	0.040	0.002	0.365	0.014
Tamoxifen		<0.000	0.912	0.563	0.785	0.856	0.002	0.984
Breed × Tamoxifen		0.002	0.001	<0.000	0.031	<0.000	0.332	0.745

^a,b,c,d^ Means within the same column carrying different superscripts are significantly different (*p* < 0.05). WG0 (W1–W0), WG1 (W2–W1), WG2 (W3–W2), WG3 (W4–W3), WG4 (W5–W4), WG5 (W6–W5), Total gain (W6–W0).

**Table 4:** The effect of breed, Tamoxifen treatments and their interactions on weekly feed intake (Means ± SE).

Groups		FI1	FI2	FI3	FI4	FI5	TFI
**Breed Effect**							
Arbor Acres		351.30 ± 9.57 ^b^	434.75 ± 9.78 ^b^	577.83 ± 15.85 ^b^	867.69 ± 12.64	920.41 ± 14.97 ^b^	3151.99 ± 22.72 ^b^
Avian48		413.48 ± 7.39 ^a^	575.27 ± 7.55 ^a^	790.13 ± 12.23 ^a^	875.81 ± 9.76	965.42 ± 11.55 ^a^	3624.78 ± 17.53 ^a^
**Tamoxifen Effect**							
Control		334.38 ± 7.53 ^b^	503.53 ± 7.69	708.34 ± 12.47 ^a^	916.75 ± 9.95 ^a^	1013.00 ± 11.77 ^a^	3483.00 ± 17.87 ^a^
TAM10		392.80 ± 12.00 ^a^	517.50 ± 12.26	709.1 ± 19.88 ^a^	863.00 ± 15.86 ^b^	927.50 ± 18.77 ^b^	3409.90 ± 28.49 ^b^
TAM20		420.00 ± 11.32 ^a^	494.00 ± 11.56	634.50 ± 18.74 ^b^	835.5 ± 14.95 ^b^	888.25 ± 17.7 ^b^	3272.25 ± 26.86 ^c^
Interaction Effect (Breed * Tamoxifen)							
Arbor Acres	Control	280.81 ± 10.96 ^d^	445.06 ± 11.2	648.19 ± 18.15 ^b^	929.06 ± 14.48 ^a^	1024.94 ± 17.13 ^a^	3328.06 ± 26.01 ^c^
	TAM10	355.60 ± 19.60 ^c^	425.20 ± 20.03	596.80 ± 32.46 ^b^	875.00 ± 25.9 ^abc^	848.80 ± 30.65 ^b^	3101.40 ± 46.53 ^d^
	TAM20	417.50 ± 17.89 ^ab^	434.00 ± 18.28	488.50 ± 29.64 ^c^	799.00 ± 23.64 ^d^	887.50 ± 27.98 ^b^	3026.50 ± 42.48 ^d^
Avian48	Control	387.94 ± 10.33 ^bc^	562.00 ± 10.56	768.50 ± 17.11 ^a^	904.44 ± 13.65 ^a^	1001.06 ± 16.15 ^a^	3637.94 ± 24.52 ^a^
	TAM10	430.00 ± 13.86 ^a^	609.80 ± 14.16	821.40 ± 22.96 ^a^	851.00 ± 18.31 ^cd^	1006.20 ± 21.67 ^a^	3718.40 ± 32.90 ^a^
	TAM20	422.50 ± 13.86 ^a^	554.00 ± 14.16	780.50 ± 22.96 ^a^	872.00 ± 18.31 ^b^	889.00 ± 21.67 ^b^	3518.00 ± 32.90 ^b^
*p-value*							
Breed		0.011	0.001	0.001	0.562	0.023	0.002
Tamoxifen		0.002	0.235	0.002	0.021	0.001	0.001
Breed × Tamoxifen		0.002	0.412	0.012	0.041	0.021	0.011

^a,b,c,d^ Means within the same column carrying different superscripts are significantly different (*p* < 0.05). FI1 (feed intake within 2nd week), FI2 (feed intake within 3rd week), FI3 (feed intake within 4th week), FI4 (feed intake within 5th week), FI5 (feed intake within 6th week) and TFI (total feed intake from 2nd till 6th week).

**Table 5:** The effect of breed, Tamoxifen treatments and their interactions on weekly feed conversion (Means ± SE).

Group		FC1	FC2	FC3	FC4	FC5	TFC
**Breed Effect**							
Arbor Acres		1.64 ± 0.08 ^a^	1.46 ± 0.08	1.79 ± 0.07	1.66 ± 0.14 ^b^	2.16 ± 0.11 ^b^	1.65 ± 0.03 ^b^
Avian48		1.49 ± 0.11 ^b^	1.52 ± 0.06	1.65 ± 0.05	2.12 ± 0.11 ^a^	2.30 ± 0.09 ^a^	1.77 ± 0.03 ^a^
**Tamoxifen Effect**							
Control		1.51 ± 0.11	1.52 ± 0.06	1.84 ± 0.06 ^b^	1.90 ± 0.11	2.15 ± 0.09	1.72 ± 0.03
TAM10		1.5 ± 0.10	1.49 ± 0.1	1.74 ± 0.09 ^ab^	1.91 ± 0.18	2.30 ± 0.15	1.73 ± 0.04
TAM20		1.63 ± 0.16	1.47 ± 0.09	1.59 ± 0.08 ^a^	1.87 ± 0.17	2.25 ± 0.13	1.67 ± 0.04
Interaction Effect (Breed * Tamoxifen)							
Arbor Acres	Control	1.54 ± 0.16	1.50 ± 0.09	2.00 ± 0.08 ^a^	1.65 ± 0.17 ^b^	2.21 ± 0.13	1.73 ± 0.04 ^a^
	TAM10	1.62 ± 0.28	1.43 ± 0.16	1.88 ± 0.14 ^ab^	1.71 ± 0.28 ^b^	2.29 ± 0.22	1.67 ± 0.07 ^ab^
	TAM20	1.76 ± 0.26	1.45 ± 0.14	1.49 ± 0.13 ^c^	1.61 ± 0.26 ^b^	1.97 ± 0.20	1.54 ± 0.06 ^b^
Avian48	Control	1.48 ± 0.15	1.53 ± 0.08	1.67 ± 0.07 ^bc^	2.14 ± 0.15 ^a^	2.08 ± 0.12	1.71 ± 0.04 ^a^
	TAM10	1.49 ± 0.20	1.55 ± 0.11	1.59 ± 0.1 ^bc^	2.11 ± 0.21 ^a^	2.31 ± 0.19	1.80 ± 0.05 ^a^
	TAM20	1.51 ± 0.20	1.49 ± 0.11	1.69 ± 0.1 ^bc^	2.13 ± 0.22 ^a^	2.53 ± 0.17	1.80 ± 0.05 ^a^
*p-value*							
Breed		0.021	0.632	0.235	<0.000	0.001	0.001
Tamoxifen		0.423	0.451	0.021	0.111	0.566	0.002
Breed × Tamoxifen		0.222	0.098	0.001	0.022	0.213	0.021

^a,b,c^ Means within the same column carrying different superscripts are significantly different (*p* < 0.05). FC1 (feed intake 2nd week / weight gain 2nd week), FC2 (feed intake 3rd week / weight gain 3rd week), FC3 (feed intake 4th week / weight gain 4th week), FC4 (feed intake 5th week / weight gain 5th week), FC5 (feed intake 6th week / weight gain 6th week) and TFC (total feed intake/ total weight gain).

**Table 6:** The effect of age, Tamoxifen treatment and sex on serological oestrogen (pg/mL) and testosterone (ng/mL) concentrations in broiler chickens (Means ± SE).

Items	Age		*p Value*	Treatment			*p Value*	Sex		*p Value*
	35	42		Control	TAM10	Tam 20		Female	Male	
Estrogen	33.95 ± 2.09 ^b^	40 ± 3.32 ^a^	0.011	38.29 ± 3.72	37.75 ± 4.06	34.14 ± 2.26	0.126	47.67 ± 1.95 ^a^	27.68 ± 1.18 ^b^	<0.000
Testosterone	0.18 ± 0.03 ^b^	0.73 ± 0.16 ^a^	<0.000	0.42 ± 0.16	0.49 ± 0.18	0.39 ± 0.11	0.258	0.07 ± 0.01 ^b^	0.72 ± 0.13 ^a^	<0.000

^a,b,c^ Means within the same row within the same category carrying different superscripts are significantly different at *p* < 0.05.

**Table 7:** Effects of interaction between age and TAM treatment and between sex and TAM treatment on serological levels of oestrogen (pg/mL) and testosterone (ng/mL) in broiler chickens (Means ± SE).

Items	Age*Treatment						*p-Value*	Treatment*Sex						*p-Value*
	35			42				Control		Tam10		Tam20		
	Control	TAM10	TAM20	Control	TAM10	TAM20		Female	Male	Female	Male	Female	Male	
Oestrogen	36.13 ± 3.74	34.17 ± 4.38	31.63 ± 3.22	41.17 ± 7.4	41.33 ± 6.94	37.5 ± 2.75	0.544	52.33 ± 2.68 ^a^	27.75 ± 2.02 ^c^	49.83 ± 3.53 ^a^	25.67 ± 1.28 ^c^	40.83 ± 2.14 ^b^	29.13 ± 2.39 ^c^	0.001
Testosterone	0.14 ± 0.05 ^c^	0.18 ± 0.05 ^c^	0.23 ± 0.06 ^c^	0.79 ± 0.33 ^a^	0.8 ± 0.33 ^a^	0.61 ± 0.23 ^b^	0.041	0.05 ± 0.01	0.7 ± 0.24	0.08 ± 0.01	0.91 ± 0.28	0.1 ± 0.05	0.61 ± 0.16	0.091

^a,b,c^ Means within the same row within the same category carrying different superscripts are significantly different at *p* < 0.05.

**Table 8:** Effects of interaction between age, Tamoxifen treatment and sex on serological concentrations of oestrogen and testosterone in broilers.

Items	Age*Treatment*Sex												
	35						42						*p-Value*
	Control		TAM10		TAM20		Control		TAM10		TAM20		
	Female	Male	Female	Male	Female	Male	Female	Male	Female	Male	Female	Male	
Oestrogen	48 ± 0.58 ^ab^	29 ± 2.3 ^def^	43.33 ± 3.38 ^bc^	25 ± 0.58 ^f^	41.67 ± 0.88 ^bc^	25.6 ± 2.16 ^f^	56.67 ± 4.1 ^a^	25.67 ± 4.1 ^f^	56.33 ± 2.91 ^a^	26.33 ± 2.73 ^ef^	40 ± 4.62 ^bc^	35 ± 3.21 ^cde^	0.017
Testosterone	0.03 ± 0.07 ^e^	0.21 ± 0.07 ^cde^	0.08 ± 0.02 ^de^	0.29 ± 0.01 ^cd^	0.1 ± 0.07 ^de^	0.3 ± 0.08 ^c^	0.07 ± 0.02 ^e^	1.51 ± 0.1 ^a^	0.08 ± 0.07 ^de^	1.52 ± 0.14 ^a^	0.1 ± 0.01 ^de^	1.13 ± 0.11 ^b^	<0.000

^a,b,c,d,e,f^ Means within the same row within the same category carrying different superscripts are significantly different at *p* < 0.05.

**Table 9:** The effect of breed, Tamoxifen treatments and their interactions on final weight, carcass weight, dressing% and relative carcass cuts percentages to carcass weight (Means ± SE).

Items		Live Weight (g)	Carcass Weight (g)	Dressing %	Thigh %	Breast %	Wings %	Neck %	Abdominal Fat %
**Breed Effect**									
Arbor Acres		1952.56 ± 35.79 ^b^	1455.67 ± 29.38 ^b^	75.20 ± 2.10 ^b^	40.12 ± 4.20 ^a^	37.03 ± 5.15 ^b^	13.13 ± 0.20 ^a^	11.03 ± 0.12	1.54 ± 0.21
Avian48		2068.17 ± 27.98 ^a^	1623.87 ± 21.9 ^a^	79.15 ± 1.42 ^a^	38.05 ± 3.15 ^b^	40.12 ± 4.25 ^a^	11.09 ± 0.12 ^b^	10.02 ± 0.23	1.43 ± 0.14
**Tamoxifen Effect**									
Control		2035.42 ± 37.96	1554.92 ± 45.38	76.17 ± 2.30	39.11 ± 4.00	38.15 ± 1.02	11.28 ± 1.33	11.45 ± 2.03	1.34 ± 0.29
TAM10		2014.78 ± 46.34	1546.39 ± 21.83	76.15 ± 4.02	38.05 ± 3.98	39.09 ± 3.00	14.00 ± 2.00	10.32 ± 1.45	1.91 ± 0.35
TAM20		1980.89 ± 32.22	1518.00 ± 47.83	76.45 ± 3.52	39..15 ± 3.90	38.10 ± 2.23	11..45 ± 1.17	11.00 ± 1.32	1.45 ± 0.27
Interaction Effect (Breed * Tamoxifen)									
Arbor Acres	Control	1944.33 ± 56.32 ^c^	1450.00 ± 74.1 ^c^	75.25 ± 3.32 ^b^	40.30 ± 3.32 ^a^	35.80 ± 8.33 ^d^	11.52 ± 3.15 ^a^	11.60 ± 4.10	1.22 ± 0.30
	TAM10	1921.67 ± 76.84 ^c^	1429.67 ± 44.85 ^c^	75.13 ± 7.04 ^b^	39.19 ± 6.05 ^ab^	38.12 ± 5.19 ^bcd^	11.60 ± 2.09 ^a^	10.32 ± 1.08	2.13 ± 0.31
	TAM20	1991.67 ± 48.45 ^abc^	1487.33 ± 42.52 ^bc^	75.19 ± 3.23 ^b^	39.67 ± 3.18 ^ab^	37.09 ± 8.23 ^cd^	11.00 ± 3.25 ^ab^	10.20 ± 4.00	1.41 ± 0.30
Avian48	Control	2126.5 ± 43.83 ^a^	1659.83 ± 52.4 ^a^	78.22 ± 2.41 ^a^	37.38 ± 4.23 ^c^	40.47 ± 5.39 ^a^	10.42 ± 2.34 ^b^	10.64 ± 3.19	1.30 ± 0.19
	TAM10	2107.89 ± 45.49 ^ab^	1663.11 ± 34.1 ^a^	79.00 ± 3.50 ^a^	37.51 ± 5.11 ^c^	40.29 ± 6.45 ^ab^	10.61 ± 2.13 ^b^	10.24 ± 3.45	1.60 ± 0.20
	TAM20	1970.11 ± 50.61 ^bc^	1548.67 ± 60.5 ^b^	79.01 ± 3.22 ^a^	38.62 ± 7.0 ^bc^	38.14 ± 10.00 ^d^	10.70 ± 3.18 ^b^	10.50 ± 3.35	1.34 ± 0.23
*p-value*									
Breed		0.001	0.004	0.011	<0.000	0.001	0.002	0.362	0.112
Tamoxifen		0.251	0.984	0.522	0.336	0.228	0.258	0.962	0.516
Breed × Tamoxifen		0.000	0.005	0.022	0.001	0.002	0.032	0.336	0.322

^a,b,c,d^ Means within the same column carrying different superscripts are significantly different (*p* < 0.05).

**Table 10:** The effect of breed, Tamoxifen treatments and their interactions on relative organ percentages based on live weight (Mean ± SE).

Items		Liver%	Intestine%	Heart%	Spleen%	Gizzard%	Comb%	Shank%
**Breed**								
Arbor Acres		2.70 ± 0.15 ^a^	5.61 ± 0.20 ^a^	0.5 ± 0.03	0.05 ± 0.01	1.50 ± 0.12 ^a^	0.02 ± 0.004	3.70 ± 0.20
Avian48		2.01 ± 0.12 ^b^	4.44 ± 0.14 ^b^	0.4 ± 0.01	0.04 ± 0.01	1.42 ± 0.13^b^	0.02 ± 0.003	3.54 ± 0.14
**Tamoxifen Effect**								
Control		2.32 ± 0.18	4.95 ± 0.21	0.40 ± 0.02 ^b^	0.04 ± 0.01	1.34 ± 0.13	0.03 ± 0.004	3.82 ± 0.18
TAM10		2.33 ± 0.15	5.12 ± 0.30	0.48 ± 0.03 ^a^	0.05 ± 0.02	1.41 ± 0.11	0.02 ± 0.005	3.60 ± 0.17
TAM20		2.50 ± 0.14	5.09 ± 0.34	0.43 ± 0.03 ^ab^	0.04 ± 0.01	1.40 ± 0.20	0.02 ± 0.004	3.52 ± 0.16
Interaction Effect (Breed * Tamoxifen)								
Arbor Acres	Control	2.51 ± 0.20 ^a^	5.2 ± 0.40 ^ab^	0.42 ± 0.02 ^b^	0.06 ± 0.02	1.40 ± 0.30	0.03 ± 0.006	4.01 ± 0.40
	TAM10	2.73 ± 0.20 ^a^	5.8 ± 0.32 ^a^	0.53 ± 0.01 ^a^	0.06 ± 0.02	1.42 ± 0.21	0.02 ± 0.003	3.72 ± 0.22
	TAM20	2.80 ± 0.12 ^a^	5.8 ± 0.41 ^a^	0.45 ± 0.04 ^ab^	0.04 ± 0.02	1.70 ± 0.22	0.01 ± 0.006	3.54 ± 0.23
Avian48	Control	2.09 ± 0.15 ^b^	4.6 ± 0.25 ^bc^	0.40 ± 0.03 ^b^	0.03 ± 0.01	1.23 ± 0.14	0.02 ± 0.004	3.52 ± 0.18
	TAM10	2.08 ± 0.14 ^b^	4.4 ± 0.30 ^bc^	0.43 ± 0.02 ^b^	0.04 ± 0.02	1.30 ± 0.20	0.02 ± 0.005	3.42 ± 0.20
	TAM20	2.10 ± 0.10 ^b^	4.1 ± 0.33 ^c^	0.41 ± 0.03 ^b^	0.05 ± 0.01	1.25 ± 0.30	0.02 ± 0.005	3.53 ± 0.21
*p-value*								
Breed		<0.000	0.014	0.007	0.663	<0.000	0.445	0.956
Tamoxifen		0.333	0.121	0.020	0.223	0.455	0.366	0.322
Breed × Tamoxifen		0.050	0.002	0.784	0.842	0.362	0.332	0.219

^a,b,c^ Means within the same column carrying different superscripts are significantly different (*p* < 0.05).
